# The Severity of Pandemic H1N1 Influenza in the United States, from April to July 2009: A Bayesian Analysis

**DOI:** 10.1371/journal.pmed.1000207

**Published:** 2009-12-08

**Authors:** Anne M. Presanis, Daniela De Angelis, The New York City Swine Flu Investigation Team, Angela Hagy, Carrie Reed, Steven Riley, Ben S. Cooper, Lyn Finelli, Paul Biedrzycki, Marc Lipsitch

**Affiliations:** 1 Medical Research Council Biostatistics Unit, Cambridge, United Kingdom; 2 Statistics, Modelling and Bioinformatics Department, Health Protection Agency Centre for Infections, London, United Kingdom; 3 Department of Health and Mental Hygiene, City of New York, New York, New York, United States of America; 4 Department of Health, City of Milwaukee, Milwaukee, Wisconsin, United States of America; 5 Influenza Division, Centers for Disease Control and Prevention, Atlanta, Georgia, United States of America; 6 Department of Community Medicine and School of Public Health, Li Ka Shing Faculty of Medicine, The University of Hong Kong, Hong Kong SAR, China; 7 Center for Communicable Disease Dynamics, Departments of Epidemiology and Immunology & Infectious Diseases, Harvard School of Public Health, Boston, Massachusetts, United States of America; George Washington University, United States of America

## Abstract

**Background:**

Accurate measures of the severity of pandemic (H1N1) 2009 influenza (pH1N1) are needed to assess the likely impact of an anticipated resurgence in the autumn in the Northern Hemisphere. Severity has been difficult to measure because jurisdictions with large numbers of deaths and other severe outcomes have had too many cases to assess the total number with confidence. Also, detection of severe cases may be more likely, resulting in overestimation of the severity of an average case. We sought to estimate the probabilities that symptomatic infection would lead to hospitalization, ICU admission, and death by combining data from multiple sources.

**Methods and Findings:**

We used complementary data from two US cities: Milwaukee attempted to identify cases of medically attended infection whether or not they required hospitalization, while New York City focused on the identification of hospitalizations, intensive care admission or mechanical ventilation (hereafter, ICU), and deaths. New York data were used to estimate numerators for ICU and death, and two sources of data—medically attended cases in Milwaukee or self-reported influenza-like illness (ILI) in New York—were used to estimate ratios of symptomatic cases to hospitalizations. Combining these data with estimates of the fraction detected for each level of severity, we estimated the proportion of symptomatic patients who died (symptomatic case-fatality ratio, sCFR), required ICU (sCIR), and required hospitalization (sCHR), overall and by age category. Evidence, prior information, and associated uncertainty were analyzed in a Bayesian evidence synthesis framework. Using medically attended cases and estimates of the proportion of symptomatic cases medically attended, we estimated an sCFR of 0.048% (95% credible interval [CI] 0.026%–0.096%), sCIR of 0.239% (0.134%–0.458%), and sCHR of 1.44% (0.83%–2.64%). Using self-reported ILI, we obtained estimates approximately 7–9× lower. sCFR and sCIR appear to be highest in persons aged 18 y and older, and lowest in children aged 5–17 y. sCHR appears to be lowest in persons aged 5–17; our data were too sparse to allow us to determine the group in which it was the highest.

**Conclusions:**

These estimates suggest that an autumn–winter pandemic wave of pH1N1 with comparable severity per case could lead to a number of deaths in the range from considerably below that associated with seasonal influenza to slightly higher, but with the greatest impact in children aged 0–4 and adults 18–64. These estimates of impact depend on assumptions about total incidence of infection and would be larger if incidence of symptomatic infection were higher or shifted toward adults, if viral virulence increased, or if suboptimal treatment resulted from stress on the health care system; numbers would decrease if the total proportion of the population symptomatically infected were lower than assumed.

*Please see later in the article for the Editors' Summary*

## Abstract 2

Marc Lipsitch and colleagues use complementary data from two US cities, Milwaukee and New York City, to assess the severity of pandemic (H1N1) 2009 influenza in the United States.

## Editors' Summary

**Background:**

Every winter, millions of people catch influenza—a viral infection of the airways—and about half a million people die as a result. In the US alone, an average of 36,000 people are thought to die from influenza-related causes every year. These seasonal epidemics occur because small but frequent changes in the virus mean that an immune response produced one year provides only partial protection against influenza the next year. Occasionally, influenza viruses emerge that are very different and to which human populations have virtually no immunity. These viruses can start global epidemics (pandemics) that kill millions of people. Experts have been warning for some time that an influenza pandemic is long overdue and in, March 2009, the first cases of influenza caused by a new virus called pandemic (H1N1) 2009 (pH1N1; swine flu) occurred in Mexico. The virus spread rapidly and on 11 June 2009, the World Health Organization declared that a global pandemic of pH1N1 influenza was underway. By the beginning of November 2009, more than 6,000 people had died from pH1N1 influenza.

**Why Was This Study Done?:**

With the onset of autumn—drier weather and the return of children to school help the influenza virus to spread—pH1N1 cases, hospitalizations, and deaths in the Northern Hemisphere have greatly increased. Although public-health officials have been preparing for this resurgence of infection, they cannot be sure of its impact on human health without knowing more about the severity of pH1N1 infections. The severity of an infection can be expressed as a case-fatality ratio (CFR; the proportion of cases that result in death), as a case-hospitalization ratio (CHR; the proportion of cases that result in hospitalization), and as a case-intensive care ratio (CIR; the proportion of cases that require treatment in an intensive care unit). Because so many people have been infected with pH1N1 since it emerged, the numbers of cases and deaths caused by pH1N1 infection are not known accurately so these ratios cannot be easily calculated. In this study, the researchers estimate the severity of pH1N1 influenza in the US between April and July 2009 by combining data on pH1N1 infections from several sources using a statistical approach known as Bayesian evidence synthesis.

**What Did the Researchers Do and Find?:**

By using data on medically attended and hospitalized cases of pH1N1 infection in Milwaukee and information from New York City on hospitalizations, intensive care use, and deaths, the researchers estimate that the proportion of US cases with symptoms that died (the sCFR) during summer 2009 was 0.048%. That is, about 1 in 2,000 people who had symptoms of pH1N1 infection died. The “credible interval” for this sCFR, the range of values between which the “true” sCFR is likely to lie, they report, is 0.026%–0.096% (between 1 in 4,000 and 1 in 1,000 deaths for every symptomatic case). About 1 in 400 symptomatic cases required treatment in intensive care, they estimate, and about 1 in 70 symptomatic cases required hospital admission. When the researchers used a different approach to estimate the total number of symptomatic cases—based on New Yorkers' self-reported incidence of influenza-like-illness from a telephone survey—their estimates of pH1N1 infection severity were 7- to 9-fold lower. Finally, they report that the sCFR and the sCIR were highest in people aged 18 or older and lowest in children aged 5–17 years.

**What Do These Findings Mean?:**

Many uncertainties (for example, imperfect detection and reporting) can affect estimates of influenza severity. Even so, the findings of this study suggest that an autumn–winter pandemic wave of pH1N1 will have a death toll only slightly higher than or considerably lower than that caused by seasonal influenza in an average year, provided pH1N1 continues to behave as it did during the summer. Similarly, the estimated burden on hospitals and intensive care facilities ranges from somewhat higher than in a normal influenza season to considerably lower. The findings of this study also suggest that, unlike seasonal influenza, which kills mainly elderly adults, a high proportion of deaths from pH1N1infection will occur in nonelderly adults, a shift in age distribution that has been seen in previous pandemics. With these estimates in hand and with continued close monitoring of the pandemic, public-health officials should now be in a better position to plan effective strategies to deal with the pH1N1 pandemic.

**Additional Information:**

Please access these Web sites via the online version of this summary at http://dx.doi.org/10.1371/journal.pmed.1000207.

- The US Centers for Disease Control and Prevention provides information about influenza for patients and professionals, including specific information on pandemic H1N1 (2009) influenza
- Flu.gov, a US government Web site, provides access to information on H1N1, avian and pandemic influenza
- The World Health Organization provides information on seasonal influenza and has detailed information on pandemic H1N1 (2009) influenza (in several languages)
- The UK Health Protection Agency provides information on pandemic influenza and on pandemic H1N1 (2009) influenza
- More information for patients about H1N1 influenza is available through Choices, an information resource provided by the UK National Health Service

## Introduction

The H1N1 2009 influenza (pH1N1) pandemic has resulted in over 209,000 laboratory-confirmed cases and over 3,205 deaths worldwide as of 11 September 2009 (http://www.who.int/csr/don/2009_09_11/en/index.html, accessed 14 September 2009), but national and international authorities have acknowledged that these counts are substantial underestimates, reflecting an inability to identify, test, confirm, and report many cases, especially mild cases. Severity of infection may be measured in many ways, the simplest of which is the case-fatality ratio (CFR), the probability that an infection causes death. Other measures of severity, which are most relevant to the burden a pandemic exerts on a health care system, are the case-hospitalization and case-intensive care ratios (CHR and CIR, respectively), the probabilities that an infection leads to hospitalization or intensive care unit (ICU) admission. In the absence of a widely available and validated serologic test for infection, it is impossible to estimate these quantities directly, and in this report we instead focus on the probabilities of fatality, hospitalization, and ICU admission per *symptomatic* case; we denote these ratios sCFR, sCHR, and sCIR respectively.

Although it is difficult to assess these quantities, estimates of their values and associated uncertainty are important for decision-making, planning, and response during the progression of this pandemic. Initially, some national and international pandemic response plans were tied partly to estimates of the CFR, but such plans had to be modified in the early weeks of this pandemic, as it became clear that the CFR could not at that time be reliably estimated [1]. Costly measures to mitigate the pandemic, such as the purchase of medical countermeasures and the use of disruptive social distancing strategies may be acceptable to combat a more severe pandemic but not to slow a milder one. While past experience [2] and mathematical models [3]–[5] suggest that between 40% and 60% of the population will be infected in a pandemic with a reproduction number similar to those seen in previous pandemics, the number of deaths and the burden on the health care system also depend on the age-specific severity of infection, which varies by orders of magnitude between pandemics [6] and even between different waves in the same pandemic [7]. Reports from the Southern Hemisphere suggest that a relatively small fraction of the population experienced symptomatic pH1N1 infection (7.5% in New Zealand, for example [8]), although these numbers are considered highly uncertain [8]. On the other hand, primary care utilization for influenza-like illness (ILI) has been considerably higher than in recent years [8], and anecdotal reports in the Southern Hemisphere have indicated that some intensive care units (ICUs) have been overwhelmed and surgery postponed due to a heavy burden of pH1N1 cases [9],[10].

The problem of estimating severity of pH1N1 infection includes the problem of estimating how many of the infected individuals in a given population and time period subsequently develop symptoms, are medically attended, hospitalized, admitted to ICU, and die due to infection with the virus. No large jurisdiction in the world has been able to maintain an accurate count of total pH1N1 cases once the epidemic grew beyond hundreds of cases, because the effort required to confirm and count such cases is proportionate to the size of the exponentially growing epidemic [11], making it impossible to reliably estimate the frequency of an event (e.g., death) that occurs on the order of 1 in 1,000 patients or fewer. As a result, simple comparisons of the number of deaths to the number of cases suffer from underascertainment of cases (making the estimated ratio too large), and underascertainment of deaths due to inability to identify deaths caused by the illness and due to delays from symptom onset to death (making the estimated ratio too small) [1]. Imperfect ascertainment of both numerator and denominator will lead to biased estimates of the CFR. Estimating the number of persons at these varying levels of severity therefore depends on estimating the proportion of true cases that are recognized and reported by existing surveillance systems. Similar problems affect estimates of key parameters for other diseases, such as HIV. In HIV, a solution to this problem—which now forms the basis for the UK's annual HIV prevalence estimates published by the Health Protection Agency [12],[13]—has been to synthesize evidence from a variety of sources that together provide a clearer picture of incidence, prevalence, and diagnosis probabilities. This synthesis is performed within a Bayesian framework that allows each piece of evidence, with associated uncertainties, to be combined into an estimate of the numbers of greatest interest [14],[15].

Here we use a similar framework to synthesize evidence from two cities in the United States—New York and Milwaukee—together with estimates of important detection probabilities from epidemiologic investigations carried out by the US Centers for Disease Control and Prevention (CDC) and other data from CDC. We estimate the severity of pH1N1 infection from data from spring–summer 2009 wave of infections in the United States. The New York City and Milwaukee health departments pursued differing surveillance strategies that provided high-quality data on complementary aspects of pH1N1 infection severity, with Milwaukee documenting medically attended cases and hospitalizations, and New York documenting hospitalizations, ICU/ventilation use, and fatalities. These are the numerators of the ratios of interest.

The denominator for these ratios is the number of symptomatic pH1N1 cases in a population, which cannot be assessed directly. We use two different approaches to estimate this quantity. In the first (Approach 1), we use self-reported rates of patients seeking medical attention for ILI from several CDC investigations to estimate the number of symptomatic cases from the number of medically attended cases, which are estimated from data from Milwaukee. In the second (Approach 2), we use self-reported incidence of ILI in New York City, and making the assumption that these ILI cases represent the true denominator of symptomatic cases, we directly estimate the ratio between hospitalizations, ICU admissions/mechanical ventilation, and deaths (adjusting for ascertainment) in New York City. Each of these two methods provides estimates for the general population, and also for broad age categories 0–4, 5–17, 18–64, and 65+ years. The result of each approach is a tiered severity estimate of the pandemic.

## Methods

### Methods Overview

**Figure 1:**
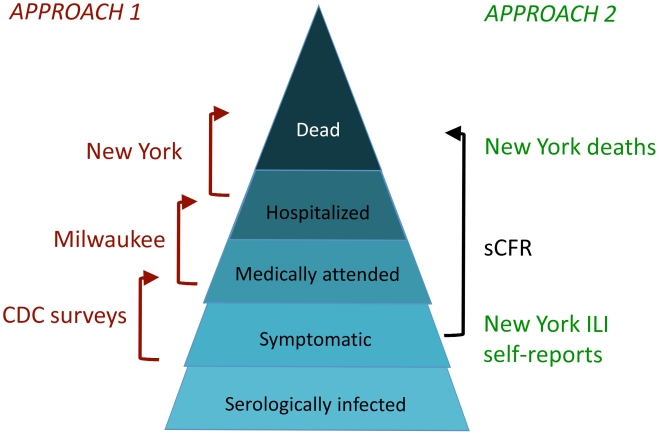
Diagram of two approaches to estimating the sCFR. Approach 1 used three datasets to estimate successive steps of the severity pyramid. Approach 2 used self-reported ILI for the denominator, and confirmed deaths for the numerator, both from New York City. Both approaches used prior distributions, in some cases informed by additional data, to inform the probability of detecting (confirming and reporting) cases at each level of severity (not shown in the diagram; see Text S1). The Bayesian evidence synthesis framework was used as a formal way to combine information and uncertainty about each level of severity into a single estimate and associated uncertainty that reflected all of the uncertainty in the inputs.

The overall goal of this study was to estimate, for each symptomatic pH1N1 case, the probability of hospitalization, ICU admission or mechanical ventilation, or death, overall and by age group. The challenge is that in any population large enough to have a significant number of patients with these severe outcomes, there is no reliable measure of the number of symptomatic pH1N1 cases. This problem was approached in two ways. Approach 1 was to view the severity of infection as a “pyramid” [16], with each successive level representing greater severity; to estimate the ratio of the top level to the base (symptomatic cases), we estimated the ratios of each successive level to the one below it (Figure 1, left side). Thus we broke down (for example) the sCFR (Figure 1, black), i.e., the probability of death per symptomatic case, into components for which data were available – the probability of a case coming to medical attention given symptomatic infection (CDC survey data); the probability of being hospitalized given medical attention (Milwaukee data); and the probability of dying given hospitalization (New York data, including a correction for those who died of pH1N1 but were not hospitalized). Approach 2 was to use the self-reported incidence of ILI from a telephone survey in New York City as the estimate of total symptomatic pH1N1 disease, and the total number of confirmed deaths in New York City as the estimate of the deaths (after accounting for imperfect ascertainment, in this case due to possibly imperfect viral testing sensitivity). In each case, prior distributions were used to quantify information on the probability that cases at each level of severity were detected; these prior distributions reflected the limited data available on detection probabilities and associated uncertainty.

All of these estimates were combined within a Bayesian evidence synthesis framework. This framework permits the estimation of probabilities for the quantities of interest (the sCFR, sCIR, and sCHR) and associated uncertainty (expressed as credible intervals [CIs]). These credible intervals appropriately reflect the combined uncertainties associated with each of the inputs to the estimate—mainly, the true numbers of cases at each level of severity, after accounting for imperfect detection—as well as the uncertainties due to sampling error (chance).

### Study Populations

Data were obtained from enhanced pandemic surveillance efforts by the City of Milwaukee Health Department and the New York City Department of Health and Mental Hygiene (DOHMH). Details of testing policies, data acquisition, and analysis are given in Text S1. All data were analyzed first in aggregate and then by age category.

### Milwaukee Data

Between April 6 and July 16, 2009, Milwaukee recorded 3,278 confirmed cases and four deaths due to pH1N1, reflecting sustained efforts to test patients reporting ILI and their household contacts from the start of the epidemic in April until mid-July. On April 27, Milwaukee initiated protocols including recommendations for testing persons with influenza symptoms and travel history to areas reporting novel H1N1 cases, using a reverse transcriptase polymerase chain reaction (RT-PCR) test specific for pH1N1. By May 7, Milwaukee issued testing guidance updated to recommend testing persons with moderate to severe symptoms, except that testing continued to be recommended for health care workers with mild, moderate or severe symptoms. We used a line list dated July 21, and in a preliminary analysis examined the frequency of hospitalization among cases by “episode date” (the earliest date in their case report). The proportion of confirmed cases hospitalized was stable around 3% up to May 20, after which it increased markedly to 6%–8% in the following weeks. We judged that this change reflected reduced testing of mild cases and limited our analysis (used to inform the ratio of hospitalizations to medically attended cases) to the 763 cases with an episode date up to or including May 20. While Milwaukee data were not the main source of estimates of ICU admission or death probabilities, we did employ hospitalized cases up to an episode date of June 14 to contribute to estimates of the ratio of deaths or ICU admissions to hospitalizations, since these should not be affected by failure to test mild cases.

### New York Case Data

New York City maintained a policy from April 26 to July 7, 2009 of testing hospitalized patients with ILI according to various criteria. These criteria evolved up to May 12, from which point they remained as follows: all hospitalized ILI patients received a rapid influenza antigen test. Those patients who tested positive on rapid test (which is known to have low sensitivity for seasonal influenza [17] and for pH1N1 [18]), and any patient in the ICU or on a ventilator, regardless of rapid test result, received RT-PCR tests for pH1N1. We obtained a line list of confirmed or probable hospitalized cases dated July 7, and found in a preliminary analysis that all patients in this line list had a date (onset or admission) in their record no later than June 30, 7 d prior to the date of the line list. Given that >90% of hospitalizations were reported in New York within 7 d, we used this entire line list without accounting for delays in reporting of hospitalizations. Also, given that 98% of admissions occurred after May 12, we did not attempt to account for changes in testing practices before May 12. This line list included a field indicating whether the patient had been admitted to the ICU or ventilated; patients were not followed up after admission to determine if this status changed. However, a chart review of 99 hospitalized cases indicated that none had been admitted to the ICU after admission, so no effort was made to account for this limitation.

Separately, we obtained a list of 53 patients whose deaths were attributed to pH1N1, of whom 44 (83%) had been hospitalized before dying. All patients with known influenza or unexplained febrile respiratory illness at the time of death had postmortem samples and/or samples taken before they died sent for PCR testing.

### New York Telephone Survey Data

To estimate levels of ILI in New York City, DOHMH conducted 1,006 surveys between May 20 and May 27, 2009, and 1,010 between June 15 and June 19. Interviews lasted 5 min and were conducted with households in both English and Spanish. The survey used a random-digit dialing (RDD) telephone sampling methodology to obtain data from a random sample of residential households in New York City. A nonrandom individual from each selected household was interviewed and provided information about all household members. Sampled numbers were dialed between five and 15 times to contact and interview a household, or until the sampled number was determined to be nonworking.

To account for this design, the data were weighted to the 2007 American Community Survey (ACS); respondents were weighted to householders by borough, age, gender, and race/ethnicity, and the population was weighted by age to the borough of residence.

The survey's RDD sampling methodology gave a useful overview of ILI in the community, but it has limitations. The design does not include individuals living in households only reachable by cellular telephone but not by a landline telephone number, and it omitted those living in group or institutional housing. Although households were randomly selected, for the sake of efficiency the interviewed adult was not. Instead, an available adult in the household provided information about all household members and themselves, which may have introduced bias. The results of the survey are being compiled for publication elsewhere. Here, we use summaries of these results by age group (see Text S1) as one means to provide denominators of symptomatic cases.

### Data on Detection Probabilities from CDC Investigations

Sources of data include two community surveys on ILI and health-seeking behavior, and two field investigations conducted during early outbreaks of pH1N1 in the US. These sources are described in further detail elsewhere [19], but are summarized here briefly. In 2007, the Behavioral Risk Factor Surveillance Survey (BRFSS), an RDD telephone survey, included a module on ILI in nine states. This module included questions to assess the incidence of ILI, health-seeking behavior, physician diagnosis of influenza, and treatment of influenza with antiviral medications during the annual 2006–2007 influenza season. In May 2009, following the emergence of pH1N1, an RDD telephone survey sampled similar to the BRFSS was conducted in the same nine states using only the ILI module from the 2007 BRFSS and limited demographic questions. In addition, some data were available from field investigations conducted during large outbreaks of pH1N1 in one community in Chicago and a university campus in Delaware. Investigations of these outbreaks consisted of household interviews in a Chicago neighborhood and an online survey of students and faculty in Delaware. These data were used to inform detection probabilities. In addition, these data were used to inform a prior distribution on the ratio between symptomatic and medically attended cases, *c_M_*_|*S*_: these surveys estimated that between 42% and 58% of symptomatic ILI patients sought medical attention [19].

### Analysis

**Figure 2:**
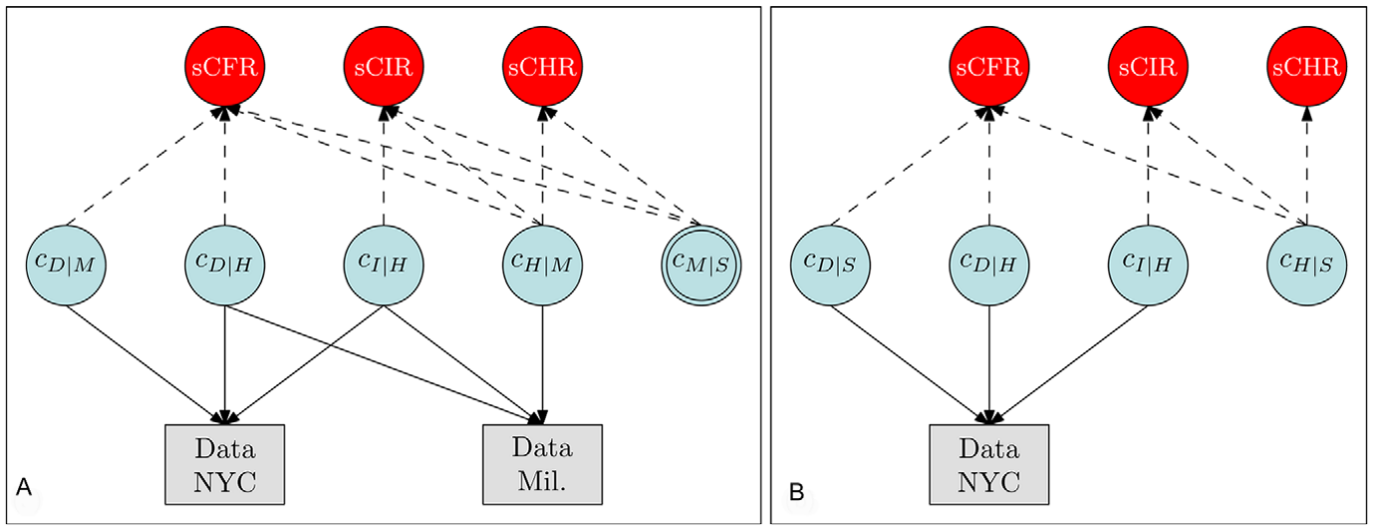
Schematic illustration of the relationship between the observed data (rectangles) and the conditional probabilities (blue circles). The key quantities of interest, sCHR, sCIR, and sCFR, are products of the relevant conditional probabilities. (A) Approach 1, synthesizing data from New York City and Milwaukee. Note that *c_M_*_|*S*_ (double circle) is informed by prior information [19] rather than observed data. (B) Approach 2, using data from New York City only, including the telephone survey. Variables: *c_D_*_|*M*_: the ratio of non-hospitalized deaths to medically-attended cases; *c_D_*_|*H*_: the ratio of deaths to hospitalized cases; *c_I_*_|*H*_: the ratio of cases admitted to intensive care or using mechanical ventilation to hospitalized cases; *c_H_*_|*M*_: the ratio of hospitalized cases to medically attended cases; *c_M_*_|*S*_: the ratio of medically attended cases to symptomatic cases; *c_D_*_|*S*_: the ratio of deaths to symptomatic cases; *c_H_*_|*S*_: the ratio of hospitalized cases to symptomatic cases.

Estimation of the probabilities of primary interest, *c_H_*_|*S*_, *c_I_*_|*S*_, and *c_D_*_|*S*_, respectively the sCHR, sCIR, and sCFR, was undertaken using a Bayesian evidence synthesis framework [14]. Details are given in Text S1, and a schematic illustration of the model is given in Figure 2. Briefly, in this framework, prior information about the quantities of interest (including the uncertainty associated with this prior information) is combined with the information coming from the observed cases at each severity level to derive a *posterior distribution* on these quantities. This posterior distribution fully reflects all information about the quantities of interest that is contained in the prior distribution and the observed data. Specifically, it was assumed that detected cases *O* at each level of severity—medically attended (*M*), hospitalized (*H*), ICU-admitted (*I*), and fatal (*D*)—represented binomially distributed samples from the true number of cases *N* at the corresponding level of severity, in the given location (New York, abbreviated N or Milwaukee, abbreviated *W*), with probability equal to the probability of detection at each level (*d*). The probability *d* for each level was informed by evidence on the probability of testing at each level of severity (which may have depended on the sensitivity of the rapid test if this was required for PCR testing) and the sensitivity of the PCR test (Table 1). Thus, for example, we defined the probability of detecting a hospitalized case in New York as *d_HN_  =  d_HN1_d_HN2_*, where *d_HN1_* was the probability of performing an RT-PCR–based test and *d_HN2_* was the sensitivity of that test. Hence, the observed number of hospitalized patients in New York, *O_HN_*, was assumed to be distributed as *Binomial*(*N_HN_,d_HN_*).

**Table 1:** Detection probabilities and their prior distributions.

Detection Probability	Components	Distributions	Rationale
*d_M_* Medically attended illness	*d_M_*_1_ probability of testing, follow-up, and reporting among medically attended patients	Uniform (0.2,0.35)	Data from CDC epi-aids in Delaware and Chicago [19]
*d_M_* = *d_M_*_1_ *d_M_*_2_	*d_M_*_2_ PCR test sensitivity	Uniform (0.95,1)	Assumption [19]
*d_HW_* Hospitalization (Milwaukee)	*d_HW1_* probability of testing, follow-up, and reporting among hospitalized patients	Uniform (0.2,0.4)	Assumption [19]
*d_HW_* = *d_HW_*_1_ *d_HW_*_2_	*d_HW_*_2_ PCR test sensitivity	Uniform (0.95,1)	Assumption [19]
*d_IW_* ICU admission (Milwaukee)	*d_IW_*_1_ probability of testing, follow-up and reporting among hospitalized patients	Uniform (0.2,0.4)	Assumption [19]
*d_IW_* = *d_IW_*_1_ *d_IW_*_2_	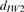 PCR test sensitivity	Uniform (0.95,1)	Assumption [19]
*d_DW_* Deaths (Milwaukee)	PCR test sensitivity and other detection	Beta (45,5)	Assumption [19] (mean 0.9, standard deviation 0.05)
*d_HW_* Hospitalization (New York City)	*d_HN_*_1_ probability of performing PCR (rapid A positive or ICU/ventilated)	0.27+0.73 (Uniform (0.2,0.71))	27% of cases were ICU-admitted so received PCR test; remainder were tested if rapid A positive, which has a sensitivity of 0.2 [17] to 0.71 (sensitivity among ICU patients in NYC)
*d_HN_* = *d_HN_*_1_ *d_HN_*_2_	*d_HN_*_2_ PCR test sensitivity	Uniform (0.95,1)	Assumption [19]
*d_IN_* ICU/ventilation (New York City)	PCR test sensitivity	Uniform (0.95,1)	Assumption [19]
*d_DN_* Deaths (New York City)	PCR test sensitivity and other detection	Beta (45,5)	Assumption [19] (mean 0.9, standard deviation 0.05)

We noted that the ratios *c_H_*_|*S*_, *c_I_*_|*S*_, and *c_D_*_|*S*_ can be built up multiplicatively from simpler components: for instance, the ratio of deaths to symptomatic infections may be expressed as *c_D_*_|*S*_  =  *c_D_*_|*H*_ *c_H_*_|*M*_ *c_M_*_|*S*_, the product of the ratios of deaths:hospitalizations, of hospitalizations:medically attended cases, and of medically attended cases:symptomatic cases. These ratios of increasing severity are similar to conditional probabilities but are not strictly so in all cases, since for example some deaths in New York City occurred in persons who were not hospitalized. For this reason we model deaths separately among hospitalized and nonhospitalized patients, i.e., *c_D_*_|*S*_  =  *c_D_*_|*H*_ *c_H_*_|*M*_ *c_M_*_|*S*_ + *c_D_*_|*M*_ *c_M_*_|*S*_. For each observed level of severity (medically attended, hospitalized, ICU, death), the true number of cases was modeled as a binomial sample from the true number of cases at an appropriate lower level, hence 
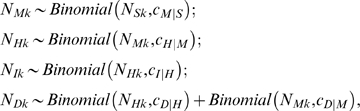
where the first subscript indicates severity and the second indicates the population (New York, Milwaukee to May 20, Milwaukee to June 14).

In Approach 1 (New York and Milwaukee data combined), for the unobserved level of severity (symptomatic cases) we used a prior distribution of *c_M_*_|*S*_ ∼ *Beta*(51.5,48.5) to represent uncertainty between 42% and 58% [19]; this distribution has 90% of its mass in this range, with a mean of 0.515. The main analysis of this first approach was performed using prior information to inform the detection probabilities. An additional “naïve” analysis was performed, in which the detection probabilities *d* were set equal to 1 at all levels of severity. Our prior distributions for the number of symptomatic cases in New York (overall and by age) were taken as ranging uniformly between zero and the proportion reporting ILI in the telephone survey (with the upper bound of that distribution itself having a prior distribution reflecting the confidence bounds of the survey results; details in Text S1). For Milwaukee, the prior distribution on symptomatic cases was taken as uniform between 0 and 25% of the population.

In Approach 2 (New York case data and telephone survey data), we made the assumption that self-reported ILI cases represented symptomatic pH1N1 infection, and used the mean and 95% confidence intervals from that survey to define a prior distribution on the number of symptomatic cases overall and by age group. We then used observed hospitalizations, ICU/ventilator use, and fatalities along with prior distributions on detection probabilities as above to inform estimates of true numbers of hospitalizations, ICU/ventilator use, and fatalities, and these in turn were used to estimate sCHR, sCIR, and sCFR.

The evidence was synthesized through a full probability model in a Bayesian framework, implemented in the OpenBUGS software [20], which uses Markov chain Monte Carlo to sample from the posterior distribution.

## Results

Table 2 shows the numbers of medically attended cases, hospitalizations, ICU admissions, and deaths in the two cities, with the Milwaukee data separated into the period (to May 20) for which we believe medically attended cases were consistently detected, and the period (to June 14) for which we consider only hospitalized cases, ICU admissions, and deaths.

**Table 2:** Cases at each level of severity.

Location	Age Group	Severity				
		Medically Attended	Hospitalized		ICU-Admitted	Dead
		to May 20	to May 20	to Jun 14	to Jun 14	to Jun 14
**Milwaukee**	0–4	126 (16%)	7 (28%)	27 (18%)	5 (20%)	0
	5–17	470 (60%)	6 (24%)	29 (20%)	7 (26%)	2 (50%)
	18–64	189 (24%)	12 (48%)	87 (59%)	14 (52%)	2 (50%)
	65+	3 (0.4%)	0	4 (3%)	1 (4%)	0
	Total	788	25	147	25	4
**New York**	**Age Group**	**Medically Attended**	**Hospitalized**		**ICU-Admitted**	**Dead (total)/Dead but not hospitalized**
	0–4	—	225 (23%)		44 (17%)	2 (4%)/2
	5–17	—	197 (20%)		51 (20%)	2 (4%)/1
	18–64	—	518 (52%)		147 (57%)	46 (87%)/6
	65+	—	56 (6%)		15 (6%)	3 (6%)/0
	Total	—	996		257	53/9

### Approach 1

We considered two alternatives to estimate the ratios of interest from the combined New York and Milwaukee data, using self-reported rates of seeking medical attention to establish the denominator. First, we obtained a naïve estimate of the ratios of deaths to hospitalizations, ignoring differences in detection across levels of severity; and second, we obtained an estimate that incorporated evidence and expert opinion on the detection probabilities at each level of severity.

The naïve estimate would suggest a median (95% CI) ratio of deaths to hospitalizations (*c_D_*_|*H*_) of 4.3% (95% CI 3.2%–5.5%), of ICU admissions to hospitalizations (*c_I_*_|*H*_) of 25% (95% CI 22%–27%), and of hospitalizations to medically attended cases (*c_H_*_|*M*_) of 3.1% (95% CI 2.0%–4.4%). The ratio of deaths outside of hospitals to medically attended cases (*c_D_*_|*M*_) is estimated to be 0.03% (95% CI 0.01%–0.06%). Incorporating the prior evidence that 42%–58% of symptomatic ILI is medically attended, this would imply a naïve estimate of the sCFR (*c_D_*_|*S*_  =  *c_D_*_|*H*_ *c_H_*_|*M*_ *c_M_*_|*S*_ *+ c_D_*_|*M*_ *c_M_*_|*S*_) of 0.081% (95% CI 0.049%–0.131%), a corresponding estimate of the sCIR (*c_I_*_|*S*_  =  *c_I_*_|*H*_ *c_H_*_|*M*_ *c_M_*_|*S*_) of 0.38% (95% CI 0.24%–0.58%), and an estimate of the sCHR (*c_H_*_|*S*_  =  *c_H_*_|*M*_ *c_M_*_|*S*_) of 1.55% (95% CI 0.98%–2.32%). If one assumes that detection probabilities are no worse at higher levels of severity than at lower levels, then these figures would be reasonable *upper bounds* on the symptomatic CFRs and CIRs.

Incorporating prior evidence of the detection probabilities at each level of severity, and thus accommodating structural and statistical uncertainties in these probabilities, we estimated that ratio of deaths to hospitalizations (*c_D_*_|*H*_) of 2.7% (95% CI 1.8%–3.8%) of ICU admissions to hospitalizations (*c_I_*_|*H*_) of 17% (95% CI 12%–21%) and of hospitalizations to medically attended cases (*c_H_*_|*M*_) of 2.9% (95% CI 1.6%–5.0%). The ratio of deaths outside of hospitals to medically attended cases (*c_D_*_|*M*_) is estimated to be 0.02% (95% CI 0.01%–0.04%).

Table 3 shows the estimates for the quantities of primary interest, overall and by age group, in the analysis that incorporated prior evidence of detection probabilities. Here, the posterior median estimate for the sCFR is 0.048% (95% CI 0.026%–0.096%) and for the sCIR is 0.239% (95% CI 0.134%–0.458%). The sCHR is estimated as 1.44% (95% CI 0.83%–2.64%).

**Table 3:** Posterior median (95% CI) estimates of the sCFR, sCIR, and sCHR, by age group, based on a combination of data from New York City and Milwaukee, and survey data on the frequency of medical attendance for symptomatic cases.

Age	sCFR	sCIR	sCHR
0–4	0.026% (0.006%–0.092%)	0.321% (0.133%–0.776%)	2.45% (1.10%–5.56%)
5–17	0.010% (0.003%–0.031%)	0.106% (0.043%–0.244%)	0.61% (0.27%–1.34%)
18–64	0.159% (0.066%–0.333%)	0.542% (0.230%–1.090%)	3.00% (1.35%–5.92%)
65+	0.090% (0.008%–1.471%)	0.327% (0.035%–4.711%)	1.84% (0.21%–25.38%)
**Total**	**0.048% (0.026%–0.096%)**	**0.239%** (0.134%–0.458%)	**1.44%** (0.83%–2.64%)

Estimates of each of these severity measures vary dramatically by age group, with the lowest severity by each measure in the 5–17 year age group. Comparing the two groups for which we have the most data, the relative risk of death for a symptomatic 18–64-year-old compared to a symptomatic 5- to 17-year-old is 15 (95% CI 5–57). The corresponding relative risks of ICU admission and hospitalization are 5 (95% CI 2–13) and 5 (95% CI 2–12) respectively. The Bayesian framework provides a natural way to estimate confidence (measured as the posterior probability) that one rate is higher than another. The probability that severity is higher in the 18- to 64-y age group than in the 5–17 age group is >99.9%, for each of fatality, ICU admission, and hospitalization respectively. The data are too sparse to say with confidence whether adults over 65 or under 65 have greater severity. For example, among the four age groups, the symptomatic case-fatality ratio is highest in the 18- to 64-y age group with posterior probability 62.%, and in those 65 and over with probability 38%. The symptomatic case-ICU admission ratio is highest in 18- to 64-year-olds with posterior probability 51% and in those over 65 with posterior probability 38%. The sCHR is highest in 18- to 64-year-olds with posterior probability 37% and in those over 65 with posterior probability 37%.

### Approach 2

Table 4 shows the estimates for the sCFR, sCIR, and sCHR, by age group, when self-reported ILI is used as the denominator for total symptomatic cases. Overall these estimates are: sCFR  = 0.007% (95% CI 0.005%–0.009%), sCIR  = 0.028% (95% CI 0.022%–0.035%) and sCHR  = 0.16% (95% CI 0.12%–0.26%). Compared to Approach 1, these estimates are nearly an order of magnitude smaller, and the age distribution differs. The relative risks for each severity in the 18- to 64-year-old group compared to the 5- to 17-year-old group are 7 (95% CI 3–25) for fatalities, 1.5 (95% CI 0.9–2.5) for ICU admissions, and 1.4 (95% CI 0.9–2.1) for hospitalizations. The CFR is highest in the 18–64 y group with posterior probability 52%. In contrast to Approach 1, the CIR is highest among 0- to 4-year-olds, with posterior probability 79%, and the CHR is highest among 0- to 4-year-olds, with posterior probability 99%.

**Table 4:** Posterior median (95% CI) estimates of the sCFR, sCIR, and sCHR, by age group, using self-reported ILI as the denominator of symptomatic cases.

Age	sCFR	sCIR	sCHR
0–4	0.004% (0.001%–0.011%)	0.044% (0.026%–0.078%)	0.33% (0.21%–0.63%)
5–17	0.002% (0.000%–0.004%)	0.019% (0.013%–0.027%)	0.11% (0.08%–0.18%)
18–64	0.010% (0.007%–0.016%)	0.029% (0.021%–0.040%)	0.15% (0.11%–0.25%)
65+	0.010% (0.003%–0.025%)	0.030% (0.016%–0.055%)	0.16% (0.10%–0.30%)
**Total**	**0.007% (0.005%**–**0.009%)**	**0.028% (0.022%**–**0.035%)**	**0.16% (0.12%**–**0.26%)**

## Discussion

We have estimated, using data from two cities on tiered levels of severity and self-reported rates of seeking medical attention, that approximately 1.44% of symptomatic pH1N1 patients during the spring in the US were hospitalized; 0.239% required intensive care or mechanical ventilation; and 0.048% died. Within the assumptions made in our model, these estimates are uncertain up to a factor of about 2 in either direction, as reflected in the 95% credible intervals associated with the estimates. These estimates take into account differences in detection and reporting of cases at different levels of severity, which we believe, based on some evidence, to be more complete at higher levels of severity. Without such corrections for detection and reporting, estimates are approximately two-fold higher for each level of severity. Using a second approach, which uses self-reported rates of influenza-like illness in New York City to estimate symptomatic infections, we have estimated rates approximately an order of magnitude lower, with a symptomatic sCHR of 0.16%, an sCIR of 0.028%, and an sCFR of 0.007%. In both approaches, the sCFR was highest in adults (in Approach 1, 18–64 y, while Approach 2 cannot distinguish whether it is higher in that group or in those 65y and older) and lowest in school-aged children (5–17 y). Data on children 0–4 and adults 65 and older were relatively sparse, making statements about their ordering more difficult. Nonetheless, these findings, along with surveillance data on the age-specific rates of hospitalization and death in this pandemic (http://www.cdc.gov/vaccines/recs/ACIP/downloads/mtg-slides-oct09/12-2-flu-vac.pdf), indicate that the burden of hospitalization and mortality in this pandemic falls on younger individuals than in seasonal influenza [21]. A shift in mortality toward nonelderly persons has been observed in previous pandemics and the years that immediately followed them [22].

These estimates are valuable for attempting to project, in approximate terms, the possible severity of a fall–winter wave of pH1N1, under the assumption that the virus does not change its characteristics. In the 1957 and 1968 pandemics, it appears that perhaps 40%–60% of the population was serologically infected, and that of those, 40%–60% were symptomatic [2],[23]–[25]. Current estimates of the transmission of pH1N1 range between about 1.4 and about 2.2, consistent with estimates of the reproduction numbers from prior pandemics [26]–[30]. To convert our estimates into population impacts, one needs to make an assumption about the attack rate and its age distribution. For each 10% of the US population symptomatically infected (with the same age distribution observed in the spring wave), our Approach 1 estimates suggest that approximately 7,800–29,000 deaths (3–10 per 100,000 population), 40,000–140,000 intensive care admissions (13–46 per 100,000 population), and 250,000–790,000 hospitalizations (170–630 per 100,000 population) will occur. These estimates scale up or down in proportion to the attack rate; for example, they should be doubled if 20% of the population were symptomatic, producing for example 15,000–58,000 deaths (6–20 per 100,000 population). Approach 2 suggests much smaller figures (for each 10% of the population symptomatic) of 1,500–2,700 deaths (0.5–0.9 per 100,000), 6,600–11,000 ICU admissions/uses of mechanical ventilation (22–35 per 100,000), and 36,000–78,000 hospitalizations (12–26 per 100,000). Again, these numbers should be scaled in proportion to the attack rate.

To date, symptomatic attack rates seem to be far lower than 25% in both the completed Southern Hemisphere winter epidemic and the autumn epidemic in progress in the US; severe outcomes seem to be considerably less numerous than those described for Approach 1 with a 25% attack rate. In New Zealand, just under 2% of the population consulted a general practitioner (GP) for ILI during the winter wave of the pandemic (http://www.moh.govt.nz/moh.nsf/indexmh/influenza-a-h1n1-update-138-180809), which is consistent with an attack rate significantly lower than 25%, though somewhat higher than the GP consultation rate observed in severe seasonal flu outbreaks such as those in 2003 and 2004 (http://www.surv.esr.cri.nz/PDF_surveillance/Virology/FluWeekRpt/2004/FluWeekRpt200444.pdf).

The level of severity estimated for the United States reflects in part the availability of antiviral treatment and other medical interventions that will not be available in all populations. Oseltamivir use was common in Milwaukee (Milwaukee Department of Health, unpublished data), and although the health care system was put under strain in both cities studied, there was no shortage of intensive care or other life-saving medical resources. In a situation of greater stress on the health system, as has been observed in certain locations in the Southern Hemisphere ([9],[10]; http://www.capegateway.gov.za/eng/your_gov/3576/news/2009/aug/185589), or in areas that lack a high-quality health care system, severity might increase in proportion to decreased availability of adequate medical attention. Worryingly, our estimates of the proportion of symptomatic cases requiring mechanical ventilation or ICU care was approximately 4–5× our estimate of the sCFR. It is possible that a substantial proportion of those admitted to ICUs could have died without intensive care. In populations without widespread access to intensive care, our results suggest that the same burden of disease could lead to a death rate 4–5× higher. Likewise, a change in the virus to become more virulent or resistant to existing antiviral drugs, or the emergence of more frequent bacterial coinfections, could increase the severity of infection compared to that observed so far.

Estimates of severity for an infection such as influenza are fraught with uncertainties [1]. Our analysis has accounted for many of these uncertainties, including imperfect detection and reporting of cases, bias due to delays between events (such as the delay from illness onset to death), and the statistical uncertainties associated with limited numbers of cases, hospitalizations, and deaths. Another major source of difficulty is the spatial and temporal variation in reporting effort for mild and severe cases; for example, most jurisdictions in the US stopped reporting mild cases on or before the second week of May, but this change varied by jurisdiction. We have attempted to avoid this difficulty by focusing on individual jurisdictions—New York and Milwaukee—for which the approach to reporting was relatively stable over time. One limitation is that Milwaukee changed its guidance during our surveillance period from testing of all symptomatic cases to testing of all symptomatic health care workers but only moderate-to-severe cases in non-health care workers. We believe that testing policies did not change dramatically during this period, because the proportion of hospitalized cases remained fairly constant; however, the sample size before this change in guidance was small. Thus, our estimates should be seen as being the risk of severe outcome among persons with symptoms, possibly biased somewhat toward those with more severe symptoms.

Despite our efforts to account for sources of uncertainty, several others remain and have not been accounted for in our analysis. First, we have assumed that for each level of severity (from medically attended up to fatal), case reporting was equal across age groups; for example, we assumed that medically attended cases were as likely to be reported for young children as for adults. It is possible that this is not the case, for example that mild cases were more likely to come to medical attention if they occurred in children than if they occurred in adults. If this were true, our conclusion that severity was higher in adults than children could be partly a result of differential reporting.

Second, the overall estimates of severity (not stratified by age group) reflect the age composition of cases in the sample we studied, especially the age composition of the lowest level of severity examined, medically attended illness. Among medically attended cases in Milwaukee, 60% were in the 5–17 y age group, the one in which severe outcomes were the least likely. A preponderance of cases within this age group may be typical of the early part of influenza epidemics, and while it has been argued that there is a shift from younger to older age groups in seasonal influenza [31] as the epidemic progresses, there is evidence, at least from the 1957 pandemic, that attack rates remain higher in children than adults throughout the course of the epidemic [2]. Since severity of pH1N1 influenza appears to be considerably higher in adults, a shift in the burden of disease from children to adults as the epidemic progresses would lead to an increase in average severity.

We note that the association between age and severity may also affect observed trends in the characteristics of cases. The World Health Organization has noted worldwide a shift from younger to older mean age among confirmed cases (http://www.who.int/csr/disease/swineflu/notes/h1n1_situation_20090724/en/index.html). If severity is lowest among children, this upward shift in age distribution may partially reflect a shift toward detection of more severe cases, rather than a true shift in the ages of those becoming infected.

Third, the symptomatic CFR, CIR, and CHR are dependent upon our estimates of the true number of symptomatic cases, N*_iSk_*, and hence are sensitive to the choice of prior distribution for these, as well as to our prior assumptions on the detection probabilities. In particular, if the probability that symptomatic patients seek medical attention and are confirmed is lower than we assume in our prior distributions, then there are more cases than are inferred by our model, and severity is correspondingly lower than our estimates. If the probability of detecting severe outcomes (hospitalizations, deaths, ICU) is lower than our prior distributions reflect, then there are more severe outcomes than our model infers, so severity is correspondingly higher.

Finally, the small sample sizes in some age groups, the over-65 year olds in particular, lead to large uncertainty in the age-specific estimates. This level of uncertainty is reflected in the wide 95% credible intervals for the estimates.

Our two approaches yield estimates that differ by almost an order of magnitude in the severity of the infection, on each of the three measures considered. How should planners evaluate these contrasting estimates? The lower estimates, using the denominator of self-reported ILI in New York City, may reasonably be considered lower bounds on the true ratios. ILI is thought to be relatively rare in May–June, hence true ILI was probably largely attributable to pH1N1 during this period in New York City. However, self-reported ILI is notoriously prone to various biases, most of which suggest that true rates are probably lower than self-reported rates. A previous telephone survey conducted in New York City found that 18.5% of New Yorkers reported ILI in the 30 d prior to being surveyed in late March 2003 [32], which represented a period of above-baseline but declining influenza activity nationally and no known influenza outbreaks in New York City [32]. The survey was repeated in October–November 2003, prior to the appearance of significant influenza activity, and 20.8% reported ILI in the 30 d prior [32]. If these surveys represent a baseline level of self-reported ILI in the absence of significant influenza activity, then the approximately 12% self-reported ILI in the telephone survey is substantially lower than this out-of-season baseline, suggesting that it likely overstates the total burden of symptomatic pH1N1 disease. The lower estimates are also broadly consistent with estimates from New Zealand, which has experienced a nearly complete influenza season [8], and from Australia (http://www.health.gov.au/internet/main/publishing.nsf/Content/cda-surveil-ozflu-flucurr.htm/FILE/ozflu-no14-2009.pdf). The higher estimates, on the other hand, were obtained using ratios of hospitalizations to confirmed medically attended cases and self-reported rates of seeking medical attention for ILI, which have been consistently measured in the range of about 40%–60%. It is possible that the special efforts of the New York City health department to identify pH1N1-related fatalities (including those not hospitalized) provides a fuller picture of the total number of deaths from this infection. Interestingly, New York City reports about the same number of hospitalizations for our study period (996) as New Zealand reports up to mid-August (972), but 3.5× as many deaths (53 versus 16) [8]. If this discrepancy reflects more complete ascertainment of deaths in New York City, it may account for much of the difference between our higher estimates of case-fatality ratios and those from New Zealand. Given the number of uncertainties cataloged above (which apply also to other jurisdictions within and outside the US), we believe that our two approaches probably bracket the reasonable range of severity for the US spring wave.

Age-specific severity patterns as estimated here are largely consistent with those one would obtain by simply comparing the incidence of confirmed cases, hospitalizations, and deaths in the US as a whole for a similar period [19], although the estimates for persons over age 65 are highly uncertain, with 95% credible intervals spanning several orders of magnitude, due to the very small number of individuals in our sample from that age group.

The estimates provided here may be compared to those for seasonal influenza. Compared to seasonal influenza, these estimates (assuming a 25% symptomatic attack rate) suggest a number of deaths in the US that could range from about half the number estimated for an average year to nearly twice the number estimated for an average year [33] (Approach 1), or a range about 10-fold lower than that (Approach 2); however, the deaths would be expected to occur in younger age groups, compared to the preponderance of deaths in persons over 65 in seasonal influenza. Such a shift in age distribution is typical for pandemics and the years that follow them [22]. Under Approach 1, and assuming a typical pandemic symptomatic attack rate of 25%, the estimated number of hospitalizations for an autumn–winter pandemic wave is considerably more than the approximately 300,000 estimated for typical seasonal influenza [34], whereas Approach 2 suggests a number between 1/3 and 2/3 of that observed in typical seasonal influenza. It should be noted that most hospitalizations, and about 90% of deaths attributed to seasonal influenza, are categorized as respiratory and circulatory, not including the more specific diagnoses of pneumonia and influenza; that is, they are due to myocardial infarction, stroke, and other proximate causes, but are nonetheless likely initially caused by influenza infection [35]. The deaths included in our study may have reflected more directly influenza-related causes and may not reflect these indirect causes of influenza-related death. Indeed, it is unclear whether the proportion of indirect respiratory and circulatory causes of death and hospitalization will be as high in this pandemic year, given the younger ages involved in most severe cases. Given these differences between the estimates here based on virologically confirmed deaths and the ecological statistical approach to estimating influenza-attributable deaths and hospitalizations for seasonal influenza, it will be difficult to interpret comparisons between the two types of estimates until (after the pandemic has finished) comparisons can be made between the ecological and the confirmed-case approach to estimating burden of hospitalization and deaths.

Our estimate of the sCFR is lower than those provided by Garske et al. [16], which ranges from 0.11% to 1.47% overall, and between 0.59% and 0.78% in the US, but which was based on confirmed plus probable (rather than symptomatic) cases. Nishiura et al. [36] estimate that between 0.16% and 4.48% of confirmed cases in the United States and Canada were fatal. Our Approach 1 includes a probability of approximately 1/8 (∼50% probability of symptomatic patients seeking care × ∼28% probability of testing and report for a symptomatic × ∼97% test sensitivity, with associated ranges for each; Table 1) to convert symptomatic into medically attended cases, and this factor accounts for most of the difference between our estimates and the earlier estimates based on confirmed or confirmed plus probable cases. Wilson and Baker [37], on the other hand, use a denominator of infections (rather than symptomatic or confirmed cases) and estimate a range of CFR from 0.0004% up to 0.6%. Our estimates fall in the middle part of this range. More recently, Baker et al. [8] used their estimates of the total incidence of symptomatic disease in New Zealand to estimate an sCFR of 0.005%, equal to the lower end of the credible interval for our Approach 2 estimate, and considerably below our Approach 1 estimate. The generally downward trend in the estimates of severity reflects early ascertainment of more severe cases (e.g., mainly hospitalized cases in the early Mexican outbreak); the authors of each of these earlier reports recognized and discussed the issue of ascertainment and its potential biasing effect on severity estimates.

While we have been careful to highlight uncertainties in the estimates of severity, our results are sufficiently well-resolved to have important implications for ongoing pH1N1 pandemic planning. The estimated severity indicates that a reasonable expectation for the autumn–winter pandemic wave in the US is a death toll less than or equal to that which is typical for seasonal influenza, though possibly with considerably more deaths in younger persons. If attack rates in the autumn match those of prior pandemics and hospitalization rates are comparable to our estimates using Approach 1, the surge of ill individuals and subsequent burden on hospitals and intensive care units could be large. However, using Approach 2, estimates of hospitalizations and ICU admissions are considerably lower. Either set of estimates places the epidemic within the lowest category of severity considered in pandemic planning conducted prior to the appearance of pH1N1 in the United States, which considered CFRs up to 0.1% (http://www.flu.gov/professional/community/community_mitigation.pdf).

Continued close monitoring of severity of pandemic (H1N1) 2009 influenza is needed to assess how patterns of hospitalization, intensive care utilization, and fatality are varying in space and time and across age groups. Increases in severity might reflect changes in the host population—for example, infection of persons with conditions that predispose them to severe outcomes—or changes in the age distribution of cases—for example a shift toward adults, in whom infection is more severe. Changes in severity might also reflect changes in the virus or variation in the access and quality of care available to infected persons.

## Supporting Information

Text S1 Supplementary methods.
(0.43 MB DOC)

## Abbreviations

BRFSS: Behavioral Risk Factor Surveillance Survey
CDC: US Centers for Disease Control and Prevention
CFR: case-fatality ratio
CHR: case-hospitalization ratio
CIR: case-intensive care ratio
CI: credible interval
DOHMH: [New York] Department of Health and Mental Hygiene
ICU: intensive care unit
ILI: influenza-like illness
pH1N1: pandemic (H1N1) 2009 [virus/influenza]
RDD: random-digit dialing
RT-PCR: reverse transcriptase polymerase chain reaction
sCFR: symptomatic case-fatality ratio
sCHR: symptomatic case-hospitalization ratio
sCIR: symptomatic case-ICU admission ratio
