# Supplementary material for: The Severity of Pandemic H1N1 Influenza in the United States, from April to July 2009: A Bayesian Analysis
Source: PLoS Med. 2009 Dec 8;6(12):e1000207. doi: 10.1371/journal.pmed.1000207 (PMC2784967; doi:10.1371/journal.pmed.1000207)
Supplement: Text S1 — Supplementary methods. (0.43 MB DOC) [file pmed.1000207.s001.doc]

**Text S1**

**Supplementary Methods:**

**The severity of pandemic H1N1 influenza in the United States, April – July 2009**

Anne M. Presanis^1^, Daniela De Angelis^1,2^, The New York City Swine Flu Investigation Team^3^, Angela Hagy^4^, Carrie Reed^5^, Steven Riley^6^, Ben S. Cooper^2^, Lyn Finelli^5^, Paul Biedrzycki^4^, Marc Lipsitch^7^

1. Medical Research Council Biostatistics Unit, Cambridge, UK

2. Statistics, Modelling and Bioinformatics Department, Health Protection Agency Centre for Infections, London, UK

3. Department of Health and Mental Hygiene, City of New York, New York, NY, USA

4. Department of Health, City of Milwaukee, Milwaukee, WI, USA

5. Influenza Division, Centers for Disease Control and Prevention, Atlanta, GA, USA

6. Department of Community Medicine and School of Public Health, Li Ka Shing Faculty of Medicine, The University of Hong Kong, Hong Kong SAR, China

7. Center for Communicable Disease Dynamics, Departments of Epidemiology and Immunology & Infectious Diseases, Harvard School of Public Health, Boston, MA, USA

**1. INTRODUCTION**

**In this Supplementary Material we provide additional details about the statistical model employed (Section 2), the data (Section 3), the detection probabilities (Section 4), the relationship between medically attended and symptomatic illness (Section 5), and the software implementation of our model (Section 6).**

**2. MODEL**

Of ultimate interest is to estimate three quantities:

1) the case-fatality ratio, defined as the ratio of the true number of H1N1pdm -attributable deaths to the true number of H1N1pdm infections; we denote this $c_{D|Inf}$because it is a conditional probability, Pr{death | infection};

2) the case-ICU admission ratio, defined as the ratio of the true number of H1N1pdm -attributable ICU admissions to the true number of H1N1pdm infections; we denote this $c_{I|Inf}$ = Pr{ICU | infection};

and 3) the case-hospitalization ratio, defined as the ratio of the true number of H1N1pdm-attributable hospitalizations to the true number of of H1N1pdm infections; this is denoted $c_{H|Inf}$= Pr{hospitalization | infection}.

Ascertainment of all infections, whether symptomatic or not, requires serological surveys, which are not yet available for H1N1pdm. Without such surveys, it is not possible to estimate the relationship between infection and more severe outcomes. We therefore attempt to estimate the ratio of severe outcomes to *symptomatic* infection: namely, the symptomatic case-fatality ratio $c_{D|S}$ = Pr{death | symptomatic infection}, the symptomatic case-ICU admission ratio $c_{I|S}$= Pr{ICU admission | symptomatic infection} or the symptomatic case-hospitalization ratio $c_{H|S}$= Pr{hospitalization | symptomatic infection}.

No jurisdiction in the United States conducted case-based surveillance for a long enough period, in a large enough population to estimate this quantity directly; in particular, jurisdictions with intensive case-finding (such as Milwaukee) did not have enough deaths or ICU admissions to make a statistically robust estimate, while jurisdictions with enough deaths and ICU admissions (such as New York) had too many cases to pursue intensive case-finding for the period over which deaths and ICU visits were registered.

Given that we do not have data on all severity levels for both locations, we aim to estimate $c_{D|S}$, $c_{I|S}$ and $c_{H|S}$, using two approaches. First, we combine data from both Milwaukee and New York on medically attended symptomatic cases, hospitalizations, ICU admissions and deaths, together with information from the Centers for Disease Control (CDC) on ascertainment probabilities and proportions of symptomatic cases seeking medical attention, to estimate the ratios $c_{D|S}$, $c_{I|S}$ and $c_{H|S}$ , assuming the conditional probabilities are equal, but age-specific, across the two jurisdictions. Second, we use data on hospitalizations, ICU admissions and deaths just from New York City, together with data on self-reported incidence of influenza-like illness (ILI) in New York, assuming that these ILI cases represent the true number of symptomatic cases, to estimate $c_{D|S}$, $c_{I|S}$ and $c_{H|S}$.

**2a. Severity Model**

**Approach 1**

We start from the simple assumption that the following hierarchy in the severity level holds: death hospital admissionmedical attendancesymptomatic case, and similarly, ICU admissionhospital admissionmedical attendancesymptomatic case, where represents inclusion. Under this assumption,

$\begin{matrix} c_{D|S}=\Pr\{\text{death}|\text{symptoms}\} \\ =\Pr\{\text{death}|\text{hospital}\}\Pr\{\text{hospital}|\text{medical attendance}\}\Pr\{\text{medical attendance}|\text{symptoms}\} \\ =c_{D|H}c_{H|M}c_{M|S} \end{matrix}$ ,

$\begin{matrix} c_{I|S}=\Pr\{\text{ICU}|\text{symptoms}\} \\ =\Pr\{\text{ICU}|\text{hospital}\}\Pr\{\text{hospital}|\text{medical attendance}\}\Pr\{\text{medical attendance}|\text{symptoms}\} \\ =c_{I|H}c_{H|M}c_{M|S} \end{matrix}$and

$$\begin{matrix} c_{H|S}=\Pr\{\text{hospital}|\text{symptoms}\} \\ =\Pr\{\text{hospital}|\text{medical attendance}\}\Pr\{\text{medical attendance}|\text{symptoms}\} \\ =c_{H|M}c_{M|S} \end{matrix}$$

where $c_{D|H}$ and $c_{I|H}$ are the probabilities of true H1N1pdm-attributable deaths or ICU admissions, respectively, conditional on true H1N1pdm-attributable hospitalizations; $c_{H|M}$is the probability of true H1N1pdm-attributable hospitalization, conditional on being a true H1N1-positive medically attended case, and $c_{M|S}$is the probability of being a true H1N1pdm-positive medically attended case , conditional on true symptomatic infection with H1N1pdm.

Clearly, these subset relations may not strictly hold. Indeed, in New York, data are available on H1N1pdm-attributable deaths which occur outside of hospital. So we instead make the assumption that

$\begin{matrix} c_{D|S}=\Pr\{\text{death}|\text{symptoms}\} \\ =\Pr\{\text{death among hospitalized}\cup\text{death among medically attended but not hospitalized}|\text{symptoms}\} \\ =\Pr\{\text{death among hospitalized}|\text{symptoms}\}+ \\ \text{ }\Pr\{\text{death among medically attended but not hospitalized}|\text{symptoms}\} \\ =c_{D|H}c_{H|M}c_{M|S}+c_{D\cap\bar{H}|M}c_{M|S} \end{matrix}$(see Figure S1).

Figure S1: Assumed severity hierarchy

We consider age-group specific values for all of these conditional probabilities so all carry a subscript $i$for age group and we denote the actual number of people who reached a given level of severity in a given jurisdiction by $N_{S}$ for symptomatic cases, $N_{M}$ for medically attended cases, $N_{H}$ for hospitalizations, $N_{I}$ for ICU admissions and $N_{D}$ for deaths. Each of these true numbers also varies by age group $i$ and location $k$ (Milwaukee, $k=W$ or NYC, $k=N$). We assume that in each age group, for each level of severity, the true number of persons at that level of severity is binomially distributed based on the corresponding conditional probability and the true number at the preceding level of severity:

(1a)

$N_{iSk}$ is given a prior reflecting our uncertainty about the number of symptomatic cases (see details in Section 5).

**Approach 2**

For the NYC only analysis, we do not consider the medically attended level, such that

$\begin{matrix} c_{D|S}=c_{D|H}c_{H|S}+c_{D\cap\bar{H}|S} \\ c_{I|S}=c_{I|H}c_{H|S} \end{matrix}$,

$c_{H|S}$is given by

(1b),

and where $N_{iP}$ is the NYC population size (considered constant), and $c_{iS|P}$are given priors, to reflect estimates from the NYC telephone survey, see section 5.

**2b. Observation model**

For a variety of reasons, detection at each level of severity will be imperfect, and thus the true values N are not observed. However, we do observe detected medically attended cases $O_{iMW}$and detected hospitalizations$O_{iHW}$ in Milwaukee, and we observe detected hospitalizations $O_{iHN}$, detected ICU stays $O_{iIN}$, and detected deaths$O_{iDN}$ in New York City. We assume that these observations $O$are related to the true numbers $N$ as follows:

(2)

where for each level of severity j, $d_{j}$ is the detection probability, i.e. the probability that a case enters our database.

**2c. Combining the models – a Bayesian approach**

Given (1a) or (1b) and (2), we wish to estimate the values of the age-specific $c_{ij}$, which can then be multiplied appropriately to estimate the age-specific (symptomatic) case-hospitalization, case-ICU and case-fatality ratios.

Figure S2 is a schematic representation of the relationship between the quantities we wish to estimate and the quantities we observe. The figure shows only a small part of the whole model in approach 1, for one generic age group and location, and for the first three levels of severity (symptomatic to hospitalized). Circles denote parameters, double circles denote parameters for which we have prior information, and squares denote observations. Solid lines denote distributional relationships and dashed lines denote functional relationships. The arrows represent the process by which the parameters, if known, would generate the data. In our case, the problem is reversed, i.e. to infer the values of the parameters given the available information. The figure provides an illustration of the flow of information from the observations and prior distributions to the unknown parameters. So for example, the information on $O_{H}$ (the observed number of hospitalizations) together with the prior on $d_{H}$ gives information on $N_{H}$, the true number of hospitalizations. Note that estimation of the parameters of primary interest (e.g. $sCHR$, the symptomatic case-hospitalization ratio) is informed indirectly by the combination of prior and sample information on intermediate but related parameters ($O_{H}$ and $O_{M}$, together with $d_{M},d_{H},N_{S}$ and $c_{M|S}$, via the true numbers $N$ and the conditional probablities $c$).

Figure S2: Simplified directed acyclic graph displaying the dependencies in part of the model.

More generally, the complete set of unknown parameters is $\underline{\theta}=(c_{ij},d_{jk},N_{ijk},sCHR,sCIR,sCFR)$, and we have observations $\underline{O}=(O_{ijk})$. Inference is then carried out in a Bayesian setting using the prior information, $P(\underline{\theta})$, and the likelihood of the observations given the parameters, $L(\underline{O}|\underline{\theta})$ to obtain, via Bayes’ Theorem, the posterior distribution $P(\underline{\theta}|\underline{O})\propto P(\underline{\theta})L(\underline{O}|\underline{\theta})$ of the parameters.

**3. DATA**

**3a. Milwaukee**

On April 27^th^, Milwaukee sent out messaging to local healthcare providers recommending testing anyone presenting with signs and symptoms characteristic of influenza (fever >100 degrees, cough or sore throat, myalgia) and travel to an area with documented H1N1. By May 7^th^ more than 100 confirmed cases had been identified, and testing guidance was updated to recommend testing persons with moderate to severe symptoms (temperature of > 101.5 and significant respiratory symptoms and significant constitutional symptoms). Testing of persons with mild symptoms was limited to health care workers. On June 15^th^ providers were told to begin testing on a fee-for-service basis. Throughout the outbreak, healthcare providers have been asked to report any suspect, probable or confirmed case of H1N1. Providers were advised about concerns regarding the accuracy of rapid flu tests and urged to use PCR as the preferred method for analysis. All confirmed and probable cases were entered into a line list on a rolling basis, and we used a line list dated July 21.

Because our primary focus for Milwaukee was on hospitalization probabilities, in a preliminary analysis we plotted the frequency of hospitalization by week of “episode date,” the earliest date (of illness onset, report or hospitalization) in an individual record. The hospitalization frequency was around 3% overall with no temporal trend up to an episode date of May 20, after which there was a dramatic upward trend in the proportion hospitalized, with 8.2%, 6.0%, and 7.0% hospitalized in the weeks that followed. We interpreted this increased hospitalization rate as evidence of declining ascertainment of mild cases, and we therefore restricted our attention to cases with illness onset date up to and including May 20. This also obviated the need to deal with censoring, as the date of the line list was two months later, far longer than the delay in reporting for nearly all cases. This created a data set of 763 cases, of whom 25 (3%) were hospitalized.

While the main source of data on the probability of ICU admission or death was New York City, such data were available, albeit in small numbers, for Milwaukee. To inform the ratio of ICU+ventilation:hospitalization and death:hospitalization, we used a larger subset of the data, on the assumption that the change in ascertainment after May 20 was due to reduced ascertainment of mild cases, not changes in ascertainment of hospitalized or more severe cases. Therefore, we considered the 147 hospitalizations with episode date up to and including June 14. Again, this was more than 30 days prior to the close of the line list, so we did not correct for censoring. Of these 147 hospitalizations, 25/147 (17%) were admitted to the ICU and/or ventilated, and 4 (3%) died.

**3b. New York City**

From April 26 to July 7, 2009, New York City maintained a policy of testing hospitalized patients with influenza-like illness (ILI) under various criteria. Criteria for testing varied up to May 12, after which point all hospitalized patients with influenza-like illness (ILI) were tested with a rapid influenza antigen test. Those patients who tested positive, and also any patient on a ventilator or in an intensive care unit (ICU) regardless of rapid test result, were tested for H1N1pdm by PCR. We obtained a line list of confirmed cases dated August 24, 2009, including 996 hospitalizations, of whom 882 had a known date of onset. Preliminary analysis indicated that >99% of hospitalizations were reported within 21 days of symptom onset. Since the last date of admission in the data set was July 6, 49 days prior to the date of the line list (August 24) ,we did not restrict this data set. Also, >97% of admissions in the data set were after May 12, so we did not attempt to account for differences in testing prior to May 12.

Separately, we obtained a list of 53 deaths attributed to H1N1pdm, of whom 44 (83%) had been hospitalized before dying. The dates of death ranged from 17 May to 19 July, but the dates of case report to New York City ranged from 13 May to 4 July; hence, these cases were all included within the time frame in which hospitalizations were being investigated. Based on the time-to-death distribution and the timing of hospitalizations, we estimated that >99.9% of deaths which would be reported from the hospitalized cases had already been reported. We therefore made no effort to account for censoring of deaths.

The data are shown in Table 1 of the main text.

**4. DETECTION PROBABILITIES**

We require information on the detection probabilities, $d_{M},d_{H},d_{I},d_{D}$. In general, these are assumed location-specific, and may consist of multiple components. We present below the evidence or prior assumptions available to inform estimates of these detection probabilities.

**4a. Detection of medically attended illness**

The detection probability for medically attended (M) illness (in Milwaukee), $d_{MW}$, may be expressed as

where $d_{MW2}$ is the sensitivity of the PCR-based tests recommended for use in Milwaukee. We assume $d_{MW1}\sim Uniform(.2,.35)$ , $d_{MW2}\sim Uniform(.95,1)$, and that the probability of censoring is 0, for the reasons described in Section 3. These assumptions are based on estimates from Reed et al. [1], using data from seasonal influenza and from Epi-Aids in Delaware and Chicago and are not Milwaukee-specific. Unlike Reed et al., we do not assume a separate probability for specimens being sent for confirmatory testing, since Milwaukee recommended against use of rapid antigen testing for screening (which would have led to false negatives and reduced detection) and since Milwaukee recommended testing of all persons with moderate to severe symptoms.

**4b. Detection of hospitalizations (Milwaukee)**

We define $d_{HW}$, the detection probability for hospitalization (in Milwaukee) as

$\begin{matrix} d_{HW}=\Pr\{\text{report hosp case}|\text{test pos\}Pr\{test pos | true hosp case already hosp and tested\}Pr\{tested | true hosp case already hosp\}} \\ =d_{HW1}d_{HW2} \end{matrix}$By using the July 21 line list but restricting analysis to cases with an episode date prior to or on May 20 (or June 14) we believe it reasonable to assume the probability of censoring is 0. We have no Milwaukee-specific data on the probability that some true hospitalizations go unreported, either because testing was not performed, or because a positive case was not reported. Hence we again follow Reed et al. in assuming

$d_{HW1}=\Pr\{\text{report hosp case}|\text{test pos\}Pr\{tested | true hosp case already hosp\}}\sim Uniform(.2,.4)$and $d_{HW2}\sim Uniform(.95,1)$ to account for imperfect PCR test sensitivity.

We assume the same priors for the detection probabilities in ICU admissions.

**4c. Detection of deaths (Milwaukee)**

$d_{DW}$ is the detection probability for deaths in Milwaukee. As with hospitalizations, we assume no censoring, since the date of the line list is a month after the last episode date in the data set we are considering (episode dates up to 14^th^ June), so that

$d_{DW}=\Pr\{\text{report death | true H1N1pdm-attributable death (already died)}\}$. We have no data to assess the probability of a death being tested for H1N1pdm, hence we assume a prior reflecting failure to detect of $d_{DW}\sim Beta(45,5)$ giving a prior mean of 0.9 and standard deviation 0.05 (a range of 0.8 – 1, as in New York, see below), covering both test sensitivity and failure to detect.

**4d. Detection of hospitalizations (New York)**

$d_{HN}$ is the detection probability for hospitalization (in New York). In New York, rapid antigen testing was used as a screen for most patients. From May 12, PCR testing for H1N1pdm was performed only on hospitalized patients who (a) tested positive on a rapid influenza A test, or (b) were in the ICU or on ventilator, regardless of their rapid influenza A status. Thus one component of $d_{HN}$ is $d_{HN1}$, the probability of PCR testing. 27% (242/909) of hospitalized H1N1pdm patients in New York were in the ICU, so for these we assume that the probability of PCR testing was 1. For the other 73% we assume the probability of PCR testing was equal to the sensitivity of the rapid test, which we model as Uniform(.2,.71). Thus we model $d_{HN1}$~.27+.73(Uniform(.2,.71)). Finally we account for imperfect sensitivity of the PCR, $d_{HN2}\sim Uniform(.95,1)$. Because of active surveillance for hospitalized cases, we assume that testing was performed as advised and was reported in all cases; hence we do not assume a separate factor for failure to test or report. As noted above, we made no effort to account for censoring of hospitalized cases.

**4e. Detection of ICU admissions, New York.**

Here we assume that detection is equal to the sensitivity of the PCR test, $d_{IN}\sim Uniform(.95,1)$, since rapid testing was not required for PCR testing. As with hospitalizations, we assume the probability of censoring is 0.

A limitation is that we only detect ICU admissions that are known by the time the hospitalized case is reported to the NYC Department of Health. Later admissions from the ward are not reported. Thus we will underestimate the proportion of ICU admissions among hospitalized cases. However, a chart review of 99 hospitalizations found that 24 (24%) were admitted to the ICU during their entire stay, a proportion indistinguishable from that in our overall dataset. Hence we conclude that this underestimation is not severe.

**4f. Detection of deaths, New York.**

New York had a policy of PCR testing all unexplained respiratory deaths involving fever. We have no data to assess the completeness of such testing. Given issues of PCR sensitivity and possible failure to test, we assume a prior distribution for ascertainment of deaths of, $d_{DN}\sim Beta(45,5)$ giving prior mean 0.9 and standard deviation 0.05), reflecting possible failure to detect H1N1pdm-attributable deaths.

**5. SYMPTOMATIC VS. MEDICALLY ATTENDED INFECTION**

We have no direct data on the number of symptomatic but not medically attended H1N1pdm infections. However, multiple epidemiological investigations have estimated the proportion of influenza-like illness that is medically attended; these estimates range from 42% to 58% [1] and include data both from prior influenza seasons and from the spring 2009 H1N1pdm influenza period. Thus we model the conditional probability of being medically attended given symptomatic infection, $c_{M|S}\sim Beta(\text{51.5,48.5)}$ giving a mean of 0.515 and standard deviation 0.05, with 90% of the probability mass between 0.42 and 0.58.

For approach 1, we also require prior distributions for the true number of symptomatic infections, $N_{S}$. For Milwaukee, we assume $N_{iSW}\sim Uniform(H_{iMW},0.25\times popn_{iW})$, i.e. a lower limit of the observed number of medically attended cases, with an upper limit of 25% of the population size. This implies a maximum clinical attack rate of 25%. For New York, we assume $N_{iSN}\sim Uniform(0,upper_{iN}\times popn_{iN})$: we have not observed medically attended cases in New York, so cannot use the observation as a lower limit. We used an upper bound of symptomatic infection in New York City based on the number of persons reporting ILI in a telephone survey conducted by the New York City Department of Health and Mental Hygiene covering a 30-day period in May-June at the height of the spring epidemic (NYC DOHMH, unpublished data):

$$\begin{matrix} upper_{0-4,N}\sim Beta(18.2,72) \\ upper_{5-17,N}\sim Beta(50.4,178) \\ upper_{18-64,N}\sim Beta(38.7,338) \\ upper_{65+,N}\sim Beta(27.6,446) \\ upper_{All,N}\sim Beta(91.4,654) \end{matrix}$$

In approach 2, the telephone survey data is used directly to inform priors for the proportion of the NYC population with symptomatic infection, rather than an upper bound:

$$\begin{matrix} c_{0-4,S|P}\sim Beta(18.2,72) \\ c_{5-17,S|P}\sim Beta(50.4,178) \\ c_{18-64,S|P}\sim Beta(38.7,338) \\ c_{65+,S|P}\sim Beta(27.6,446) \\ c_{All,S|P}\sim Beta(91.4,654) \end{matrix}$$

**6. IMPLEMENTATION**

The Bayesian model described in Section 2 used the data and priors as presented in Sections 3 to 5, and was implemented in the OpenBUGS software. This uses Markov chain Monte Carlo to obtain samples from the posterior distributions of the parameters of interest. Three chains of 1,000,000 iterations each were run, starting from different initial values. Summary statistics were based on the last 200,000 iterations of each chain, after discarding the first 800,000 as a burn-in period.

Convergence for the quantities of primary interest which were reported in the main text, the conditional probabilities $c_{ij}$, was assessed both visually and using Gelman-Rubin-Brooks plots and we are satisfied the chains converged for these in most age groups. In approach 1, the probability of hospitalization given medical attendance did not reach quite the same level of convergence as the other $c_{ij}$, particularly for the 65+ age group. This is due to the paucity of data available for this ratio: only data from Milwaukee is available, up till May 20^th^, the period for which ascertainment of mild cases was assumed constant over time. The observed numbers of hospitalizations in particular are very small, with 0 hospitalizations observed in the 65+ age group. This has a knock-on effect on the true numbers of medical attendances and symptomatic infections ($N_{iSk}$ and $N_{iMk}$) , so that their Markov chains also did not quite reach the same level of convergence as the chains for the true numbers of hospitalizations, ICU admissions and deaths.

The posterior estimates for the symptomatic case-fatality, case-ICU admission and case-hospitalization ratios are reliant on the estimates of $N_{iSk}$, the true number of symptomatic cases, and are hence sensitive to the choice of prior. Convergence for $N_{iSk}$ improves as the upper limit for its prior is reduced, i.e. as the maximum clinical attack rate becomes smaller. However, it would not be reasonable to assume a maximum clinical attack rate of less than the telephone survey estimates for New York or less than 25% for Milwaukee, given our lack of prior knowledge on these. For this reason we do not report estimates of the total number symptomatic. Despite the uncertainty, there is some information available in the likelihood to update the estimates of the number symptomatic: the posteriors do not simply reflect the prior distributions (Figures S3 and S4).

In approach 2, we are satisfied that the chains converged for the conditional probabilities $c_{ij}$ in all age groups.

Figure S3: Prior versus posterior number of symptomatic infections, Approach 1

Figure S4: Prior vs posterior number of symptomatic infections, by age, Approach 1

**Reference**

1. Reed C, Angulo F, Swerdlow D, Lipsitch M, Meltzer M, et al. (2009) Estimating the burden of pandemic influenza A/H1N1 -- United States, April-July 2009. Emerg Infect Dis. In press. DOI: 10.3201/eid1512.091413
